# Development of a point-of-contact technique to measure adenosine triphosphate: A quality improvement study

**DOI:** 10.1016/j.amsu.2019.03.013

**Published:** 2019-04-05

**Authors:** Janet Pierce, John B. Hiebert, Diane Mahoney, Qiuhua Shen, Jill Peltzer, Faith Rahman, Samantha Johnson, John T. Pierce

**Affiliations:** University of Kansas Medical Center, School of Nursing, Mail Stop 4043, 3901 Rainbow Blvd, Kansas City, KS, 66160, USA

**Keywords:** Adenosine triphosphate (ATP), Point-of-contact, Energy, Fatigue

## Abstract

**Purpose:**

Patients with heart failure with preserved ejection fraction (HFpEF) experience fatigue due to impaired myocardial bioenergetics. Cardiomyocyte function depends on the delivery of adenosine triphosphate (ATP), yet there is no convenient bedside method to measure ATP. The purpose of this study was to develop a point-of-contact measurement of ATP that can be used in a clinical setting.

**Methods:**

In a laboratory setting, digital finger punctures were conducted using 5 μl and 10 μl of capillary blood placed into various amounts of water (H2O). After mixing the solution for 10 s, a Hygiena AquaSnapTM Free ATP probe was placed into the solution for 10 s for the detection of ATP. The probe was then placed into the Hygiena luminometer for 15 s, and a value in relative light units (RLU) was obtained.

**Results:**

Test samples using 10 μl of blood diluted from 50 to 500 mls of H2O produced ATP readings of 10,000-7569 RLUs. Using 5 μl of blood in 375–900 ml of H2O decreased the ATP values to 6459-4189 RLUs. Dilutional volume sparing experiments were conducted with ATP standards to determine the concentration of ATP per RLUs.

**Conclusion:**

Patients with HFpEF have increased metabolic demand and impaired myocardial bioenergetics. Thus, identifying a method to measure ATP that is quick and accurate is imperative to accurately assess cellular energy production in this population. Point-of-contact measures, such as ATP, are needed for precision-guided treatment. Data from this study provides the first step toward developing evidence for health policies related to managing fatigue.

## Highlights

• Adenosine triphosphate (ATP) is required for almost all cells to produce energy.
• A point-of-contact instrument is needed in the clinical setting to measure ATP.
• To assess the symptom of fatigue, a measure of ATP is preferred.
• Using blood from a finger stick, an ATP measurement can be obtained in 2 min.

## 1 Introduction

The production of adenosine triphosphate (ATP) is vital for cell function, and 90% of ATP is produced in the mitochondria [1]. Food molecules are metabolized for valuable energy in the form of ATP. With aging, increased mitochondrial free radicals lead to a decrease in the respiratory chain complexes and reduced ATP production. This increase in free radicals also occurs with various diseases such as heart failure and diabetes. Decreased ATP production can lead to many pathophysiologic conditions and cellular apoptosis [2], thus an accurate method for measuring ATP production is important to determine a patient's cellular function.

There are commercially available rapid ATP detection devices for food service, healthcare, and water quality. For this study, we used the Hygiena EnSURE™ ATP luminometer instrument and swabs designed for measuring ATP water quality. The AquaSnap™ Free swab has a honey-comb shaped dipper that extracts ATP from water that is then mixed with a reagent and placed into the device. Within 2 min, light is emitted in direct proportion to the amount of ATP and displayed in relative light units (RLU).

It is well known that muscle fatigue is related to mitochondrial dysfunction and/or decreased production [3,4]. Fatigue is a common subjective symptom in which patients often report feelings of tiredness, lack of energy, or exhaustion. The etiology is multifactorial and associated with numerous causes such as anemia, mitochondrial dysfunction, hypoglycemia, and neurological disorders. Fatigue may be experienced by individuals in different dimensions as physical, mental, and emotional tiredness [5]. This perceptual state has been closely related to ATP production [6].

One potential cause of the depletion of ATP is mitochondrial dysfunction. Thus, the reduction in ATP production could produce fatigue-like symptoms in patients [7]. This is observed particularly in patients with heart failure with preserved ejection fraction (HFpEF) [8]. Currently, in the clinical setting there is no point-of-contact method used to obtain a quantitative measure of ATP. This is problematic because the healthcare provider has no measure to assess fatigue other than a questionnaire when patients express that they have increased or decreased fatigue. Thus, the project aim of this study was to adapt an ATP instrument for a point-of-contact testing that could be used in the clinical setting among patients with heart failure as a reliable measurement of fatigue.

## 2 Methodology

### 2.1 Location, sample, and design

This protocol was reviewed by the University of Kansas Medical Center (KUMC) Human Subjects Committee and was designated as a quality improvement study to develop a point-of-contact technique to measure ATP. The study procedures were completed in a laboratory at KUMC. Experiments were conducted to determine the optimal amount of blood and water and the technique to obtain reliable and valid measures of ATP. After the method and protocol were developed, they were tested on 12 healthy adult volunteers.

### 2.2 Materials and method

We used a commercially available Hygiena EnSURE™ ATP luminometer instrument with an AquaSnap™ water testing device that is a self-contained ATP test to monitor ATP levels in water. The AquaSnap™ Free swab only measures dissolved ATP outside of living cells (non-microbial ATP). This instrument can detect down to 0.01 fmol of ATP. The advanced photodiode sensor technology of the swab is not affected by drops or shakes, and the swabs are reliable for 15 months at refrigerated temperatures of 2–8 °C or 4 weeks at room temperature (21–25 °C).

After attempted blood sampling with different pipettes and locations for digital punctures, we established the optimal method. This included wrapping a heating pad around the subject's index finger for 2 min. The heating pad was removed, and the lateral surface of the first upper extremity phalangeal digit (index finger) was cleaned with alcohol pad for 10 s. The Accu-Check® softclix lancet device was pressed against the finger and then released and the finger was milked to encourage a small bleb of blood. Using a pipette with a cut tip, we obtained the blood sample.

**Fig. 1:**
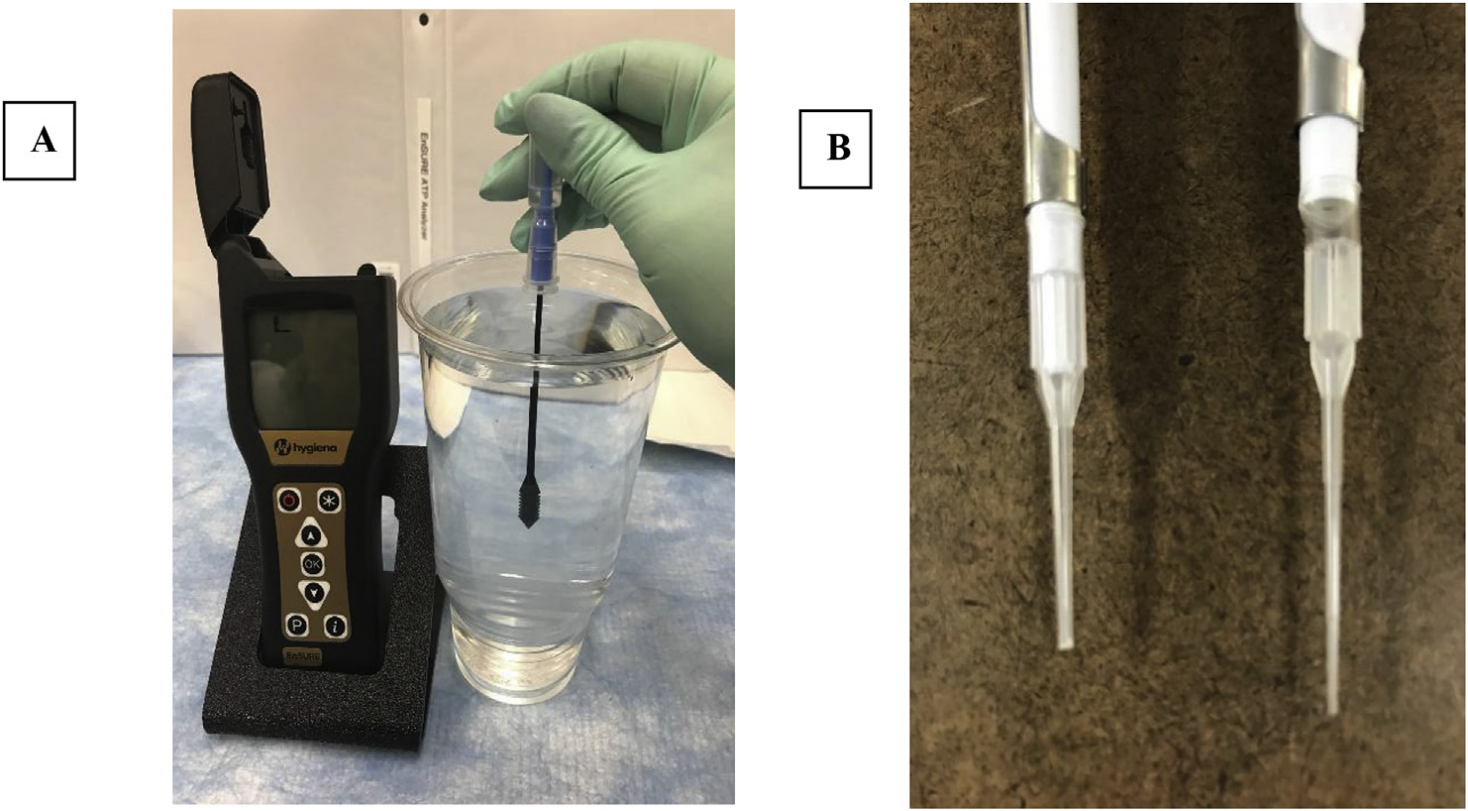
A. Hygiena EnSURE™ ATP luminometer instrument with the AquaSnap™ FREE swab. B. Cut and regular pipette tip tested for blood sampling.

The first set of experiments in the laboratory was conducted to determine a method for sampling ATP. We tested 10 μl and 5 μl blood samples in various volumes of water (H_2_O) using the Hygiena EnSURE™ ATP luminometer instrument with the AquaSnap™ FREE swab. We found that using an automatic pipette with the tip cut provided an adequate sample of 5 μl of blood from a finger puncture. The sample was then placed in 900 ml of H_2_O and mixed for 10 s. The AquaSnap™ swab was then placed in the solution for 10 s and then inserted into the Hygiena EnSURE™ ATP instrument. After 15 s, the instrument displayed an ATP value in relative light units (RLU) (Fig. 1).

The second experiment focused on establishing the concentration of ATP. For this experiment, we purchased a 300 μl standard solution containing 10 mM of ATP from Sigma Aldrich (Burlington, MA). We placed 50 μl of a standard ATP solution (10 mM) into 5 ml of saline to produce a stock solution (10 mM/ml ATP). Next, a 500 μl of the ATP stock solution was placed in 5 ml of saline to produce a 10 μM/ml ATP solution. Using the Hygiena Ensure ATP Luminometer, we placed an AquaSnap™ Free swab into the solution to detect ATP within 15 s. Further sequential dilutions were conducted with ATP standards to determine the concentration of ATP per RLU. We weighed five AquaSnap™ Free swabs and then placed the swabs into water for 10 s to determine the amount of solution the swab absorbed.

## 3 Results

From one subject, two samples of 10 μl of blood in 900 ml of H_2_O resulted in no detectable values because the concentration was too high to detect ATP with this instrument. Next, 5 μl of blood was placed in 400 ml, 600 ml, and 800 ml of H_2_O without a cut pipette tip and the values ranged from 3507 to 6459 RLU (Table 1).

**Table 1:** Laboratory testing of fluid and blood volumes with the Hygiena Ensure ATP Luminometer.

Sample	Sample 1 (RLUs)	Sample 2 (RLUs)
10 μl of whole blood in 900 ml of H_2_O (tip intact)	Over range^a^	Over range^a^
5 μl of whole blood in 400 ml of H_2_O (tip intact)	6459	5365
5 μl of whole blood in 600 ml of H_2_O (tip intact)	5557	4853
5 μl of whole blood in 800 ml of H_2_O (tip intact)	4891	3507

a No detectable value of ATP, RLUs = Relative Light Units.

For the next set of experiments, we collected 3 samples from 2 subjects using cut pipette tips to test if the values would be more reliable. With a cut pipette tip, 5 μl of blood in 250 ml of H_2_O produced values for subject one from 9410 to 9451 and for subject 2 from 9509 to over 10,000. Using 5 μl of blood in 900 ml of H_2_O (cut tip), the values ranged from 4536 to 4589 for subject 1 (mean = 4,569) and 5841 to 5855 (mean = 5,849) in subject 2 (Table 2).

**Table 2:** ATP values (RLU) from blood samples of 2 subjects using 250 ml and 900 ml of H_2_O.

	Subject 1			Subject 2		
	1	2	3	1	2	3
5 μl of whole blood in 250 ml of H_2_O - cut tip	9428^a^	9410^a^	9451^a^	9509^a^	9514^a^	no value
5 μl of whole blood in 900 ml of H_2_O - cut tip, different pipette	4536^a^	4589^a^	4581^a^	5851^a^	5855^a^	5841^a^

a Relative Light Units (RLU).

The sequential ATP dilutions were conducted using 10 mM/ml, 10 μM/ml, 10 nM/ml, 1 nM/ml, 0.5 nM/ml, and 0.1 nM/ml. In the first three dilutions, the ATP concentration was too high (>10,000 RLU) to detect with the instrument. Once the dilution was at 1 nM/ml, the instrument detected a value of 8223 RLU. For a dilution of 0.5 nM/ml, the instrument detected a value of 5230 RLU. At a dilution of 0.1 nM/ml, the instrument detected a value of 1530 RLU (Table 3).

**Table 3:** Serial dilutions to determine ATP concentrations.

ATP Concentrations	RLU Results
10 mM ATP (stock solution)	
↓	
10 μM ATP	Over limit (>10,000 RLU)
↓	
10 nM ATP	Over limit (>10,000 RLU)
↓	
1 nM ATP	8,223
↓	
0.5 nM ATP	5,230
↓	
0.1 nM ATP	1,530
↓	
No ATP in saline (Blank)	0

*Relative Light Units (RLU).

**Fig. 2:**
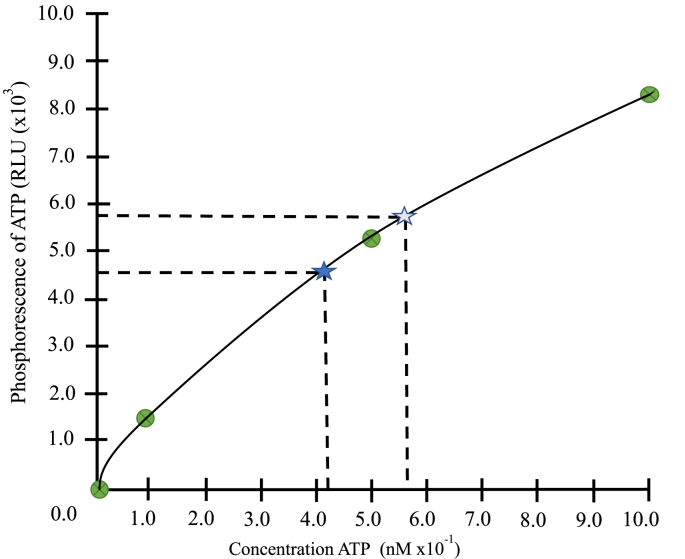
Phosphorescence of ATP in RLUs for known concentrations of ATP. RLU = Relative Light Units. = 0 nM, 0.1 nM, 0.5 nM, 1 nM ATP. = mean 4,569. = mean 5,849.

**Fig. 3:**
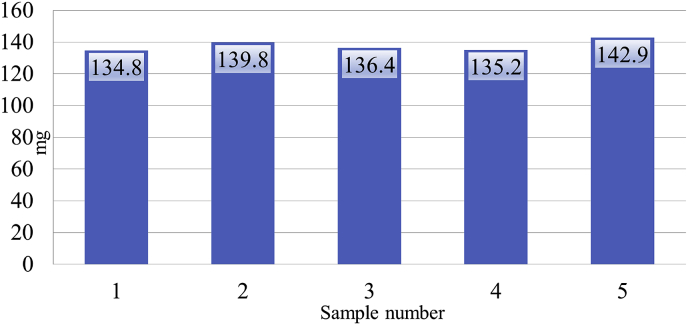
Weight of samples collected by the AquaSnap™ Free swab.

Fig. 2 illustrates the phosphorescence of ATP in RLUs for known concentrations of ATP (1 nM ATP, 0.5 nM ATP, 0.1 nM ATP, and 0 nM ATP). The stars represent mean values of three samples (Table 1) from 2 subjects when 5 μl of whole blood was placed in 900 ml of H_2_O using the Hygiena Ensure ATP Luminometer. The weight of absorption of H_2_O from the five swabs that were tested ranged from 134.8 mg to 142.9 mg with a mean of 137.8 mg (Fig. 3).

In 12 healthy adults ranging in age from 31 to 71 years, we obtained more than 10 μl of blood from the lateral side of the first upper extremity phalangeal digit. Applying a heating pad before the digital puncture required less milking of the finger. We also found that cutting the pipette tip before sampling allowed for more accurate collection and release of the blood into the water before testing for ATP.

## 4 Discussion

The goal of this study was to develop a method to adapt the Hygiena Ensure ATP Luminometer for use as a point-of-contact instrument to measure ATP in a clinical trial or clinical setting. The EnSURE™ instrument is currently being used to measure ATP from surfaces and liquid samples in hospitals, recreational areas, and the food industry. It is ideal to use in a clinical setting because it provides real-time results in 15 s and is portable [9,10]. We established a method to obtain an adequate blood sample from a capillary puncture and place it in a large fluid volume to acquire an ATP value in RLU within 15 s. However, we did not know the ATP concentration for the values displayed in RLU. Thus, we performed a series of dilutions with known concentrations of ATP and determined the ATP concentration in our samples were between 1 and 0.1 nM.

Adequate ATP is important for cellular function, and ATP deficiency is associated with numerous diseases including impaired cardiac function leading to congestive heart failure [11]. Brain tissue function is dependent on ATP, and ATP deficiency impacts the occurrence and clinical course of diseases such obstructive sleep apnea [12] and Alzheimer's disease [13]. The role of ATP in cellular processing of sugars is critical in diseases such as diabetes [14].

Individuals with various diseases often report fatigue and clinicians and researchers often measure the patient's fatigue with a questionnaire [15,16]. However, there are many factors that can affect a patient's response when completing a questionnaire such as the presence of a family member, cultural norms, or personality type [17]. Thus, a measure of ATP along with the data from a fatigue questionnaire would provide an improved assessment of an individual's fatigue. We are now using this technique with the Kansas City Cardiomyopathy questionnaire to measure fatigue in patients with HFpEF in a clinical setting.

Almost all living cells contain the ATP molecule [18], and therefore a direct measure of biological concentration is possible. For years ATP has been quantified by measuring the light produced by the firefly enzyme luciferase using a luminometer. The amount of light produced is directly proportional to the amount of ATP [17,[19], [20], [21]]. A common method employed to quantify ATP production is to isolate mitochondria and measure ATP using fluorometry [22]. This very accurate and well-defined method is very time consuming and cannot be completed in a clinical setting [23]. Thus, the Hygiene monitoring instrument was developed for rapid and reliable measures of ATP for surfaces, water, microorganisms, and humans. The AquaSnap™ FREE swab has a bulb with a liquid stable luciferase product designed to eliminate the need to reconstitute lyophilized pellets and help provide precise results of low levels of ATP [10,24]. After testing various amounts of water and blood samples, we determined that adding 5 μl of blood to 900 ml water and using the Hygiene ATP instrument we detected ATP in the 5000 and 4000 RLU range. With the dilutional experiments, we determined that the concentrations of ATP for this RLU ranged between 1 and 0.1 nM of ATP.

## 5 Conclusion

For our research with HFpEF and other diseases that are related to decreased ATP, we needed to find a measure of ATP that could be completed rapidly and accurately in a clinical setting. We have adapted the Hygiena AquaSnap™ equipment to be used as a point-of-contact instrument in our clinical trial in patients with HFpEF to measure ATP. Using 5 μl of blood in 900 ml of H_2_O appears to be the ideal amount to obtain accurate and reliable ATP values. We have developed a time-sensitive protocol to measure ATP in RLU that are in nanomole concentrations. While the instrument displays values in RLU and not ATP concentration, these experiments have helped us to determine the ATP concentrations of a sample when the range is between 3,500 and 6,000 RLU.

## Provenance and peer review

Not commissioned, externally peer reviewed.

## Ethical approval

This work has been approved by the University of Kansas Medical Center Institutional Review Board as Quality Improvement study.

## Sources of funding

Supported by the Department of Health and Human Services, National Institutes of Health, National Institute on Aging (Grant Number: 1R01AG054486-01A1).

## Author contribution

Pierce, J. D: Study concept and design, data collection, data analysis, writing the paper, reviewing and editing the paper.

Hiebert, J: data collection, data analysis, writing the paper, reviewing and editing the paper.

Mahoney, D: data collection, writing the paper, reviewing and editing the paper.

Shen, Q: writing the paper, reviewing and editing the paper.

Peltzer, J: writing the paper, reviewing and editing the paper.

Rahman, F: figure and table design, referencing, reviewing and editing the paper.

Johnson, S.: figure and table design, reviewing and editing the paper.

Pierce, J. T: design, data collection, data analysis, reviewing and editing the paper.

## Conflicts of interest

No conflict of interest to declare.

## Research registry number

ClinicalTrials.gov Identifier: NCT03133793.

## Guarantor

Pierce, J.

Hiebert, J.

Mahoney, D.

## References

[1] Kuhlbrandt W. (2015). Structure and function of mitochondrial membrane protein complexes. BMC Biol..

[2] Drew B., Leeuwenburgh C. (2003). Method for measuring ATP production in isolated mitochondria: ATP production in brain and liver mitochondria of Fischer-344 rats with age and caloric restriction. Am. J. Physiol. Regul. Integr. Comp. Physiol..

[3] Karatzaferi C., Adamek N., Geeves M.A. (2017). Modulators of actin-myosin dissociation: basis for muscle type functional differences during fatigue. Am. J. Physiol. Cell Physiol..

[4] Wan J.J., Qin Z., Wang P.Y., Sun Y., Liu X. (2017). Muscle fatigue: general understanding and treatment. Exp. Mol. Med..

[5] Donovan K.A., Stein K.D., Lee M., Leach C.R., Ilozumba O., Jacobsen P.B. (2015). Systematic review of the multidimensional fatigue symptom inventory-short form. Support. Care Canc..

[6] M N.R., Armstrong C.W., Lewis D.P., Butt H.L., Gooley P.R. (2015). Metabolic profiling reveals anomalous energy metabolism and oxidative stress pathways in chronic fatigue syndrome patients. Metabolomics.

[7] Filler K., Lyon D., Bennett J., McCain N., Elswick R., Lukkahatai N., Saligan L.N. (2014). Association of mitochondrial dysfunction and fatigue: a review of the literature. BBA Clin..

[8] Banerjee P. (2017). Heart failure: a story of damage, fatigue and injury?. Open Heart.

[9] Bhatia V., Gupta A., Sharma S., Shandil R., Wadhawan M., Agrawal N., Kumar A. (2017). Residual contamination and bioburden after reprocessing of single-use endoscopic ultrasound needles: an ex vivo study. Dig. Endosc..

[10] Renaud D.L., Kelton D.F., LeBlanc S.J., Haley D.B., Jalbert A.B., Duffield T.F. (2017). Validation of commercial luminometry swabs for total bacteria and coliform counts in colostrum-feeding equipment. J. Dairy Sci..

[11] Bhatt K.N., Butler J. (2018). Myocardial energetics and heart failure: a review of recent therapeutic trials. Curr. Heart Fail. Rep..

[12] D'Rozario A.L., Bartlett D.J., Wong K.K.H., Sach T., Yang Q., Grunstein R.R., Rae C.D. (2018). Brain bioenergetics during resting wakefulness are related to neurobehavioral deficits in severe obstructive sleep apnea: a 31P magnetic resonance spectroscopy study. Sleep.

[13] Cai Q., Tammineni P. (2017). Mitochondrial aspects of synaptic dysfunction in alzheimer's disease. J. Alzheimer's Dis..

[14] Flemming N.B., Gallo L.A., Forbes J.M. (2018). Mitochondrial dysfunction and signaling in diabetic kidney disease: oxidative stress and beyond. Semin. Nephrol..

[15] Gorman G.S., Elson J.L., Newman J., Payne B., McFarland R., Newton J.L., Turnbull D.M. (2015). Perceived fatigue is highly prevalent and debilitating in patients with mitochondrial disease. Neuromuscul. Disord..

[16] Kouijzer M., Brusse-Keizer M., Bode C. (2018). COPD-related fatigue: impact on daily life and treatment opportunities from the patient's perspective. Respir. Med..

[17] Deng N., Guyer R., Ware J.E. (2015). Energy, fatigue, or both? A bifactor modeling approach to the conceptualization and measurement of vitality. Qual. Life Res..

[18] Mendelsohn B.A., Bennett N.K., Darch M.A., Yu K., Nguyen M.K., Pucciarelli D., Nelson M., Horlbeck M.A., Gilbert L.A., Hyun W., Kampmann M., Nakamura J.L., Nakamura K. (2018). A high-throughput screen of real-time ATP levels in individual cells reveals mechanisms of energy failure. PLoS Biol..

[19] Branchini B.R., Southworth T.L. (2017). A highly sensitive biosensor for ATP using a chimeric firefly luciferase. Methods Enzymol..

[20] Branchini B.R., Southworth T.L., Fontaine D.M., Kohrt D., Welcome F.S., Florentine C.M., Henricks E.R., DeBartolo D.B., Michelini E., Cevenini L., Roda A., Grossel M.J. (2017). Red-emitting chimeric firefly luciferase for in vivo imaging in low ATP cellular environments. Anal. Biochem..

[21] Morciano G., Sarti A.C., Marchi S., Missiroli S., Falzoni S., Raffaghello L., Pistoia V., Giorgi C., Di Virgilio F., Pinton P. (2017). Use of luciferase probes to measure ATP in living cells and animals. Nat. Protoc..

[22] Fisher-Wellman K.H., Davidson M.T., Narowski T.M., Lin C.T., Koves T.R., Muoio D.M. (2018). Mitochondrial diagnostics: a multiplexed assay platform for comprehensive assessment of mitochondrial energy fluxes. Cell Rep..

[23] Santangelo M.F., Libertino S., Turner A.P.F., Filippini D., Mak W.C. (2018). Integrating printed microfluidics with silicon photomultipliers for miniaturised and highly sensitive ATP bioluminescence detection. Biosens. Bioelectron..

[24] Bill C., Danielson J.A., Jones R.S. (2017). Salivary intercellular adenosine triphosphate testing in primary caretakers: an examination of statistical significance versus diagnostic predictability. Clin. Exp. Dent. Res..

